# Continuous Knowledge Translation in Action: Designing a Programmatic Research Trial for Equitable Eye Health for Rural Nepalese Women

**DOI:** 10.3390/ijerph17010345

**Published:** 2020-01-03

**Authors:** Yadira Perez Hazel, Cathy Malla, Anita Afford, Tessa Hillgrove, Reeta Gurung, Anjila Dahal, Sarita Shah, Mohan Krishna Shrestha, Anu Manandhar

**Affiliations:** 1 Fred Hollows Foundation, Melbourne, VIC 3053, Australia; tessa.hillgrove@gmail.com; 2 Fred Hollows Foundation, Darwin, NT 0810, Australia; AAfford@hollows.org; 3 Tilganga Institute of Ophthalmology, Kathmandu 44600, Nepal; reetagurung@gmail.com (R.G.); sarita.shah@tilganga.org (S.S.); mohan.shrestha@tilganga.org (M.K.S.); anu.manandhar@tilganga.org (A.M.); 4 Fred Hollows Foundation, Kathmandu 44600, Nepal; adahal@hollows.org

**Keywords:** eye health, equity, gender, women, Nepal, programmatic design process, pragmatic trial, continuous knowledge translation

## Abstract

Reaching vulnerable populations through programmatic eye health interventions requires a focus on not only the intervention strategies, but the adaptability of the program design process itself. Knowing who is left behind and why solutions that will be effective on the ground at the time of implementation are not necessarily generated. There is a need for eye health programmatic design processes that can trial interventions and allow for continuous knowledge translation along the way. In rural Nepal, women are impacted by multiple and interconnected determinants of health, as well as unique barriers to accessing information and services, requiring targeted programming strategies. This article describes a programmatic design and knowledge translation process that aims to increase women’s uptake of eye health services in rural Nepal. The article outlines key learnings of this knowledge translation process, and how this may contribute to addressing gender equity in eye health.

## 1. Introduction

Reaching those who are left behind is pivotal to reduce avoidable blindness. The Sustainable Development Goals have called on governments and non-government organizations (NGOs) to identify and address inequity, both globally and nationally. In terms of eye health, globally, women account for 55% of visual impairment [1] and face unique barriers in access to eye care services, and therefore have lower rates of utilisation of these services [2]. For example, women in low- and middle- income countries are much less likely to receive cataract surgery than men [3].

The Global Gender Gap Report 2018 ranks Nepal 105 out of 149 countries [4] in terms of equity for women and men in four areas: health, education, economy and politics, and this gender disparity is also seen in utilisation of eye health care services [5]. In Nepal, women have a higher prevalence of blindness than men (2.7% compared to 2.1%) [6] but are more likely to report difficulty in accessing eye health services. There is low utilisation amongst females of eye health services relative to the eye disease burden, particularly in rural and hard-to-reach areas. The cataract surgical coverage for women is lower than that of men (82.9% compared to 87.6%) [6] suggesting that women drop out of the referral pathway and are less likely to receive surgery that they need. Women who require spectacles are also less likely to have them than men (44.6% compared to 58.2%) [7]. Several studies have identified reasons relating to low access for eye health care for women in Nepal, including low levels of health literacy amongst females, low decision making power of women within the family, the health care of women and girls being a lower priority within the family, a lack of social and financial freedom to travel for health care, and inadequate gender-sensitive services provided close to where women live [8,9]. Women in rural areas of Nepal face particular additional challenges compared to those residing closer to urban facilities, including the geographical structure of Nepal that means for some women travelling for eye care is extremely difficult [10,11,12]. These findings echo women’s experience in other low- and middle-income settings [13]. 

In response, programmatic interventions in Nepal have aimed at increasing access to eye care amongst women, through provision of gender sensitive information in eye health facilities, outreach camps close to people’s homes, subsidised surgeries, mobilisation of female community health volunteers, and community awareness activities. These strategies have had varying levels of success in terms of increasing the proportion of women accessing eye care services in Nepal [14]. Women and men can have different understandings of and experiences with eye health and eye health services [9,13]. Therefore, responding to and respecting beneficiaries’ differing needs and identities can be one way that health services can attempt to increase uptake of services, in line with a patient-centred approach [15,16]. There is a need for flexible and responsive program designs that are informed by local knowledge and allow for elucidation of what actually works in the field to increase access and uptake of eye care services amongst women in rural Nepal. Additionally, there is a need for studies that allow for a deep understanding of why certain strategies may or may not be successful, in accordance with a gender equity focus.

Gathering data on what strategies work, and why, requires a nuanced understanding of local factors on the ground during any intervention, including any socio-economic and political forces occurring. A programmatic intervention that is designed with rigorous and agile data collection, and continuous and point in time knowledge translation can enable this. Such a study requires a design that allows for gender-related factors to be unpacked in an ongoing way, in order to understand the layers of barriers to access from a localized gender perspective. Research processes can aim to promote equity in access to health care services through investigating how gender and other dimensions, such as poverty and geography, intersect and result in certain patterns of how people may or may not access health services [17]. Using knowledge translation processes in an integrative and participatory way can promote discussion and reflexive practices that can aim to reduce inequities [18,19]. The aim of this article is to outline key learnings of this knowledge translation process, and how this may contribute to addressing gender equity in eye health. It also aims to provide key learnings from our focused attention to the knowledge translation process as part and parcel of a multi-layered approach to increasing equity for rural women in Nepal. The setting for this research is in Dadeldhura, Baitedi, and Kailali distrcits of Nepal.

## 2. Materials and Methods

**Figure 1:**
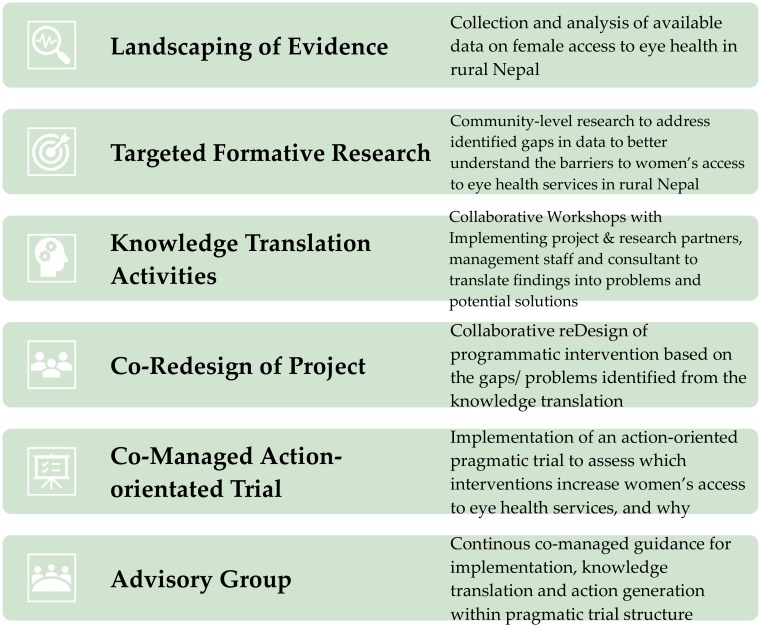
Continuous knowledge translation process.

A continuous knowledge translation process was undertaken to design and implement a series of research and programmatic intervention activities. Figure 1 shows the workflow of this process, which was developed by documenting the step-by-step process that occurred over three years. These steps were not planned in advance but came about as evidence was generated, and decisions were collectively made about what the next step should be. 

### 2.1. Landscaping of Evidence

The Fred Hollows Foundation (FHF) has a long-standing 26-year partnership with Tilganga Institute of Ophthalmology (TIO), based in Kathmandu, Nepal. As part of the non-government community that provide eye care services in Nepal, TIO and FHF have a long history of collaboration to alleviate avoidable blindness through TIO’s Nepal Eye Program, both in Nepal and internationally, and have worked together to implement both hospital-based and outreach programs across Nepal. This work includes advocating for increased government support for providing public eye health services. TIO and FHF were both concerned that the prevalence rates of cataract among women and the low relative uptake of treatment for cataract signaled that the current models of working could do more to reach women, particularly in rural areas of Nepal. To identify knowledge gaps in this area, desk-based research was carried out by a team consisting of TIO and FHF project staff to ascertain what was known about female access to eye health in Nepal, barriers and facilitators, and interventions that had been trialed both in Nepal and in other parts of the world. To address this, FHF and TIO planned a programmatic intervention that utilized methods of reaching rural women based on the program teams’ knowledge and experience of what may work. However, before this project was implemented, the team decided that they needed some more in-depth knowledge about the barriers faced by these women in accessing eye care services, in order to tailor the interventions. At this point, it was determined by TIO and FHF project staff that it would be beneficial to have some more specific data on the experiences of women requiring eye care and the barriers and challenges to accessing screening and treatment in the proposed project intervention districts, as this level of detail was lacking in the available evidence. 

### 2.2. Targeted Formative Research

In order to address this knowledge gap, TIO and FHF designed a formative research project to better understand the barriers to women’s access to eye health services in rural Nepal. The formative research project included (a) a large community survey (qualitative and quantitative) and key informant interviews that sought to understand community knowledge, attitudes and practices (KAP survey) around eye health services, (b) a quantitative surveys of general community members, people who had undergone cataract surgery, and people who have been recommended for cataract surgery but hadn’t undergone surgery, in order to uncover barriers and facilitators to access to eye care (c) a desk-based health policy analysis, seeking to identify entry points to facilitate women’s access to eye health services. The research was undertaken by an external research consultant group. All three methodologies were guided by the following research objectives: to identify and describe areas of eye care disadvantage, in particular for women; to assess the level of knowledge, attitudes and practices of community members engaging in eye care; to understand and describe contributors to access inequities; to identify opportunities for intervention to address inequity, and to identify indicators and data collection approaches that will allow for ongoing monitoring or equity of access. This research was carried out in nine of 22 project implementing districts representing all three ecological regions and four development regions of Nepal, in order to assess any differences between regions in terms of access to eye care. (The results of this formative research will be published elsewhere).

### 2.3. Knowledge Translation Activities

**Figure 2:**
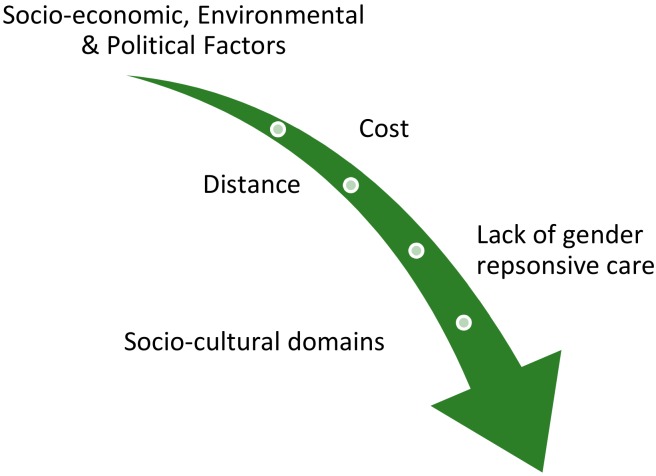
Social Determinants of Health Domains from Formative Research.

Once the formative research was completed, an initial knowledge translation workshop was held with the research consultants and FHF research advisor, FHF Nepal team and TIO research, program and executive management team members, with the aim of presenting the results of the research and exploring gaps in the analysis to ensure the FHF and TIO programs team had enough data for decision-making. Participants asked the consultant clarifying questions about the research methodology, results, and recommendations. A second knowledge translation workshop was held including the FHF Nepal team, TIO programs and research team, and FHF technical advisors in program design, gender and research. The main goal of this workshop was the exploration of the root causes of the barriers identified by the research in relation to women accessing eye health, and the brainstorming of possible solutions. The participants read through the formative research results together to establish a group understanding of the key themes arising from the data. The group then organized responses by each research question, combining and aligning the qualitative with the quantitative results from a range of sources. The group then created emerging thematic areas, triangulating data where possible from across qualitative and quantitative data, as well as across sources. Some of these were related to knowledge, attitudes, and practices (for example, beliefs around cataract surgery) and some to clinical data, which the group attempted to bring together in order to develop emerging themes from broader social determinant domains (see Figure 2). 

### 2.4. Co-Redesign of Project

During the second knowledge translation workshop, the participants each took a theme and generated a problem statement such as “women have less access to resources within the family to pay for health care.” A brainstorming activity was conducted which sought to bring to the surface ideas around the root causes of the problems and its effect on access to eye health, in order to create a ‘problem tree’. A second brainstorming activity was conducted to start generating possible programmatic responses to these problems, drawing from experience and knowledge of programmatic strategies that have been successful in other contexts, such as eye health programs in other countries [20], or other health projects in Nepal [21]. The group made decisions on which of these assumptions were going to be tested within the project and a project logic was developed, including draft project outputs and outcomes. 

Through these two knowledge translation workshops, the group identified that the initial plan for a programmatic intervention (developed before the formative research had been undertaken) would not address many of the root causes of the barriers to access. While the interventions planned in the original intervention would make services more inclusive and accessible to women, it was apparent that these strategies would not affect several significant issues that limited women being aware of and able to reach services in the first place. The group made a decision to go back to the drawing board and re-work the program design, to ensure that a program aiming to improve women’s access to eye health services would be addressing the main barriers to their participation. 

### 2.5. Co-Managed Action-Oriented Trial

Once the decision had been made to go back and re-design the programmatic intervention, the FHF research team conducted a brief literature review to explore the evidence for effective strategies to address the core barriers identified in the research in the Nepal or other comparable contexts (in particular the barriers of cost and transport). There was very limited evidence of successful strategies in the peer-reviewed literature. This presented an opportunity for the program. Given the lack of evidence on “what works”, the TIO and FHF Nepal team were keen to explore contextually appropriate, innovative solutions to the barriers. They decided to do this within the structure of a pragmatic trial, and generate evidence on how barriers to access could be overcome (and to estimate how much it would cost to address them). The FHF Nepal team and TIO programs team communicated to their program partners and other service providers to determine their interest in participating in the pragmatic trial. With initial interest confirmed from eye health hospitals operating in rural areas of Nepal, FHF, TIO, implementing partners, and technical advisors developed a pragmatic trial research design. 

**Figure 3:**
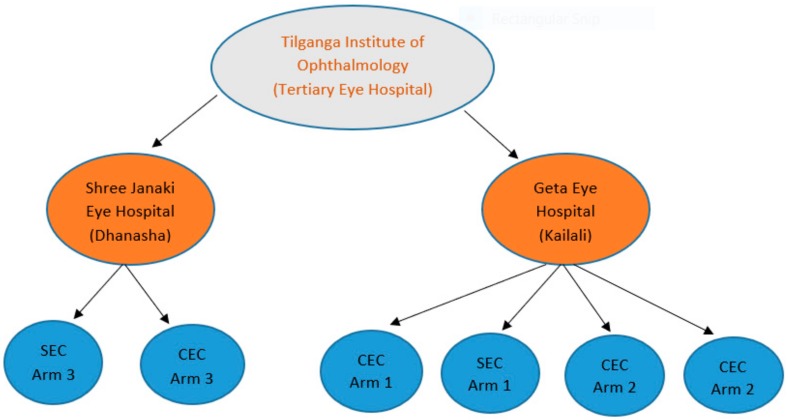
Pragmatic Trial Design. Tilganga Institute of Ophthalmology is a tertiary eye hospital and also a managing partner of the project. Shree Janaki Eye Hospital and Geta Eye Hospital are base, partner hospitals of the project situated in East and West part of Nepal. The trial included one Secondary Eye Centre (SEC) and one Community Eye Centre (CEC) under Shree Janaki Eye Hospital—control site. Geta Eye Hospital was the intervention site and included three Community Eye Centers and one Secondary Eye Centres. The CEC and SEC from both base hospitals are situated in plain and mountain part of Nepal.

The design comprised of three different trial arms (see Figure 3, with participating eye health hospitals, secondary eye centers and community eye centers allocated to either control or intervention arm groups. The study featured a multiple-baseline-across-settings approach, which meant services allocated to the control group would only implement gender-sensitive programming 12 months after the other sites had commenced. The pragmatic trial was designed as a project implementation investigation whereby different strategies for overcoming barriers to accessing eye health services for rural women in Nepal were trialed in different communities and hospitals. The team focused on designing a project that provided the rigor needed to record evidence of the programmatic process and results that could inform present and future programming in Nepal and broader FHF programming about what works (and what did not work well) to improve gender equity in access and uptake to eye health services. During the design process of the pragmatic trial, the FHF research team sought consultation with an external research group (CERA) to provide advice into the trial’s design, including sample size calculation support. 

### 2.6. Advisory Group

Part of the design of the pragmatic trial was the creation of a ‘Project Advisory Group’ (PAG). The purpose of the group was to maintain oversight of the project and to provide strategic guidance for the implementation investigation component of the project, thereby reducing the risk that program implementation decisions would be undertaken without considering implications for the pragmatic trial. Membership comprised TIO project & research staff (implementing partners), FHF Nepal program staff (managing partners), management staff and technical expertise staff from both organizations. The group met virtually every second month, or more frequently as specific issues arose. The specific responsibilities of the group were:

- To give strategic guidance on the research element of the project including design, data collection tools and analysis, dissemination and advocacy.
- Provide support for any adjustment to the programmatic activities resulting from trial findings.
- To give overarching support to the implementation team with respect to contracting research partners and coordinating trial activities with programmatic activities.
- To provide guidance on implementation of the project including coordination of programming activities to ensure the project is meeting milestones.

The PAG’s first meeting occurred during the protocol and tool development workshop which provided opportunity for in-depth design and solution-oriented programming to occur collaboratively. Subsequent meetings were coordinated by implementing partner staff who also created the agenda driving the discussion towards developing solutions for on-the ground programmatic challenges. 

Once the pragmatic design was confirmed by all partners, a consultant was hired with experience implementing research into programmatic projects, to incorporate a research methodology for data collection and analysis. A three-day co-designing workshop was held with PAG members, the research consultant and the full research team (including TIO and FHF members). The workshop included knowledge sharing activities, protocol review and finalisation, development of evaluation tools, and discussion of the process and logistics of carrying out each element of the intervention. There was also an opportunity to begin drafting the ethics application together. The results of this pragmatic trial will be published forthcoming. 

### 2.7. Development of Key Learnings

The key learnings were developed by first four authors through review and analysis of themes that emerged from PAG meetings notes and semi-structured informal interviews with knowledge translation activity coordinators and participants. The meeting notes included programmatic updates that included implementing partners context, challenges, successes and recommendations as well as the solutions offered by PAG and decision-making rationales. Once themes were developed, the lead authors shared a draft with project stakeholders including implementing partners for feedback and incorporated recommendations into key learnings.

## 3. Results

This continuous knowledge translation process has allowed for a deep exploration of the complexity of implementing gender equity strategies into an eye health intervention in rural Nepal. This section will outline the key learnings as the results. There were a number of key learnings from this process, which will be useful in the design and delivery of future eye health programmatic interventions. These key learnings are outlined below: 

**It was important not just to have information about gaps and solutions, but to test what the barriers and facilitators are for addressing these gaps and implementing these solutions in the field, in real time.** We found that the formative research around knowledge, attitudes and practices, and quantitative data around who accessed services, was useful in order to uncover gaps in access to eye care for women, and to make some assumptions about what programmatic interventions could potentially alleviate these gaps. However, we couldn’t be sure that these interventions would be successful. Through setting up a pragmatic trial, we were able to test these assumptions in a more rigorous way than usual programmatic monitoring and evaluation, as we were able to set up tools, processes and dedicated human resources to collect and analyse targeted data from the different project trial areas. Our findings showed that at each level of intervention, there was a range of gender-related barriers and facilitators that needed to be explored, and then the interventions were refined within the parameters of the design. With the PAG guiding the project implementation, we were able to tackle gender equity barriers and enablers as they arose within the programmatic trial.

**The flow from each step in the process to the next was important in order to unravel the complexities of gender equity programming.** Initially, we designed a gender and eye health project that included strategies that had been utilized in other contexts and that we assumed would increase access to eye care for women. Discussion between key stakeholders prompted the realization that we needed more definitive information on specific barriers in the project locations, which led to the development of the formative research. We believed that the formative research would fill our knowledge gap in terms of the structural and other local barriers that women were facing in order to access eye care services in these particular regions. Part of the formative assessment was an extensive literature review that outlined both challenges and interventions tried in Nepal. We understood that the “innovation” in tackling inequity was not in figuring out the perfect design at the start but to consider how to imbed learning and redesign in throughout the process. During the knowledge translation activity for the formative research, we decided to design a programmatic process where we would test evidence-based activities, figure out what worked and what didn’t, and then use this place-based knowledge to promptly refine our interventions at point of service. The knowledge translation workshop therefore provided a structured process to not only translate formative research into a program design over two days, but to allow the group to come to a realization that we needed a similar knowledge translation process throughout the intervention process. This approach resulted in a complete redesign of the project, which was possible partly due to the flexibility of and strategic commitment to equity by the donor (Department of Foreign Affairs and Trade (DFAT) through the Australian NGO Cooperation Partnership—ANCP) and organisational evidence-based programmatic processes that allowed the FHF and TIO program teams to take a few more weeks to redesign in light of new evidence/information and to integrate continuous “learning and pivoting” throughout the project through the stewardship of the PAG. 

**This process has allowed a deeper conversation about ‘doing gender work’ within our organisation.** There is the risk in eye care programming that addressing barriers for women to eye health care becomes something that may be addressed through identifying an isolated issue and solving that. For example, if transport is a barrier, then programs should support women to afford and access transport. Such an approach is a gender sensitive approach that tends to identifies an isolated issue and brings that issue to stakeholders (including beneficiaries) to develop solutions such as the provision of transport subsidies for women. However, in order to work towards a gender transformative approach, it is important to assess issues systemically uncovering the deeper, underlying structural issues, for example, examining what are the systematic gendered reasons why women access transport less than men, and then developing solutions to challenge stereotypes and change behavior. The PAG meetings had a standing agenda that included programmatic updates from implementing staff and problem-solving discussions. The programmatic updates outlined the challenges and noted the issues & potential solutions from the staff and beneficiaries’ point of view. A key approach to these PAG problem-solving sessions was that all members provided their feedback and outlined the rationale behind their recommendations creating transparency of cultural, professional and experiential knowledge being draw on and transforming the meetings into knowledge translation activities. 

**Increasing explicit gender consciousness among funders and implementing partners is essential.** Facing and exploring the challenges that arose during implementation was important, because it meant that we were exploring these gender equity-related issues more deeply, and also challenging our own gender equity assumptions. Gender is so deeply rooted in ourselves and the way we organize our societies and daily lives that, unless we stop and critically reflect on these beliefs, we will not recognize our own biases and how these biases affect program design and implementation [22]. Problem solving and reflection was an on-going activity within this entire process, and we built in check-points that were situation and time based where problem solving could occur. This involved ongoing and clear communication between the project coordinator and the PAG. 

**The process benefited through being co-designed from problem identification, solution design through to continuous evidence translation.** An important element of the process was that the pragmatic trial was designed by a collaborative, multi-disciplinary team with a diverse range of skills, experience and contextual knowledge including the research advisor, the technical specialist (gender advisor with program development skills), a relatively new FHF country team-managing partner (with programming and research skills and commitment to equity), TIO program team-implementing partner with years of programming experience in Nepal, and Regional Program Coordinator (with strong knowledge translation, research, and program development skills) who all worked together to both unpack the research and develop the pragmatic trial approach. This approach also allowed for accountability over the process and the project development, especially when trialing an approach that requires continuous knowledge translation and action. The co-design process, with all team members involved in knowledge translation process, re-design, and ongoing management through the PAG, contributed to a capacity development process for all involved, with most team members articulating this growth in capacity in research design and management, gender equity, and working in the country context during project progress report development. This would not have occurred to the same extent if the activities and design process was more siloed. 

**The process must be co-managed and involve the right stakeholders.** Having a PAG with local experts and international research and gender technical expertise allowed a diverse skill set and world views within the group, so that compromises were made, and problems solved together. Everybody needed to have an opportunity to have their voice heard and feel like their opinion was being respected, and therefore this was a space where all ideas, challenges and successes were openly discussed and solutions developed together. This was key to moving the project forward, allowing a space to discuss how to address gender equity issues that arose within project implementation. For example, there were some restrictions on the women hired for a gender focal role at the implementing hospital, in not being able to travel for project implementation activities, and it was suggested by the implementing hospital that a male gender focal person needed to be hired and trained in gender responsive training in order to carry out the day long trips of community engagement. The sex of the gender focal person was not predetermined in the pragmatic trial program implementation plan, but it highlighted an example of women’s reality within a workplace in Nepal. At the PAG meetings, this challenge was openly discussed, better understood and solutions developed, at the same time challenging the personal assumptions of members of the PAG. Co-design and co-management of these processes increased trust among the project teams. It was important for all of us to be involved in the design process, that it was collaborative and evolved with peoples’ input. 

**Gender equity work can be difficult and time-consuming.** We need to acknowledge that this work requires human resources oriented to do equity work in public health and commitment to relationship-building as well as appropriate funding, and this needs to be accounted for. As highlighted by the challenges that the PAG had to constantly negotiate, all members of this group were taking on this role that required continuous engagement throughout the project rather than just at the beginning and end of the project. Additionally, the program design team worked through the challenges of balancing program implementation alongside a pragmatic trial. If our project’s aim is to challenge and contribute to changing systemic gender inequities it cannot just be a ‘tick-box’ as part of a project with some targeted ‘gender’ activities within a project. 

These insights may be useful for others doing similar work, to take into consideration when designing interventions around equity. 

## 4. Discussion

Avoidable blindness remains a major public health problem in Nepal. The prevalence of blindness is higher in women than men, yet women are accessing cataract surgeries at a lower rate than men. 

The focus on equity work within international development has been treated as a problem that can be solved by changing the content while keeping the same model of programming. In doing so, public health interventions risk exacerbating gender inequities [23] in serving all, for tackling inequities requires an understanding that inequities exist because barriers exist. Eye care research and service delivery has some way to go in terms of reducing ‘gender blindness’ in research and service delivery [13]. Given how addressing gender inequity could improve the health of women and girls (and men and boys), eye health programs, policies, research and practices need to continue developing evidence on the impact of gender inequity on community health, establish processes and policies that support gender equity and offer interventions that explicitly model the relationships, collaborations and practices needed to create gender equity (such as eye and primary health workforce). Gender equity requires that processes and outcomes improve health and work to change negative gender norms at the same time. The increased investment has the potential to transform health outcomes by potentially improving the lives of millions worldwide [24].

We found that utilising a pragmatic trial design was beneficial in allowing the space to unpack gender-related project challenges as they arose. Being able to trial different approaches at a small scale, utilising a research approach so that we could be systematic about data collection, was an effective way of learning at a micro-level about gender-related issues, and testing solutions one-by-one. The use of a pragmatic trial was made with an awareness of the needed evidence in designing project interventions that not only respond to local needs, but to take an approach that could potentially be replicated in other FHF projects that focus on gender equity. Having a PAG allowed us to be able to delve deeply into how gender intersects within project implementation and activities, and we could therefore identify the gender related challenges and develop solutions that looked at the root cause of these challenges. In this way, the PAG allowed for these continuous knowledge translation processes to occur. 

Issues of inequity are contextual, power-driven, at times historical, and affected politically by national and geo-political activities. These forces call for “interventions” to act locally while linking globally. There are no ‘quick programmatic fixes’ to gender equity work. Identifying the problem doesn’t mean that the issue is resolved. This process resembles Masuda et al. [18] concept of an approach to knowledge translation which “recognizes the power relations that underpin policy decisions that arise when certain ‘‘accepted realities” are promoted over others and introduces a reasonable process for understanding and shifting “how we know” so that a more equitable and just reality can be pursued. (459)” The pragmatic trial provided the opportunity to build smaller scale, time-bound evidence-based interventions while using the implementation challenges that arose on the ground as opportunities for evidence translation and activity pivots that could lead to increased uptake of eye health services for women living in rural Nepal. 

The women’s issue related to accessing eye care is multifaceted [2,20]. Often, the issues identified by formative assessments are broad and community level. The issues related to using eye health services are deeply interlinked with social, cultural, environmental and economic issues. Programming that seeks to reach women left behind by current health systems and interventions require the capability to shift processes and funding according to on the ground realities and social relationships [25]. Women’s experience is intimately linked to that of men’s and thus the work of providing gender-responsive health services to women requires (a) an understanding of women’s needs, (b) their relationship to men and community, and the multiple ways that their needs and their relationship to others and their environment transforms. The experience of women are not all the same, and other barriers due to ethnicity, disability, place of residence need to be considered if we want to ensure access to eye health for those most in need. 

Having a PAG allowed us to be able to delve deeply into how gender intersects within project implementation and activities, and we could therefore identify the gender related challenges and develop solutions that looked at the root cause of these challenges. In this way, the PAG allowed for these continuous knowledge translation processes to occur. Equity work requires the development of effective and transformative co-management models that respect local staff’s knowledge, experience and capacity while also providing training, mentoring, and capacity-building activities. Co-management models seeks to bring capable teams together with skills that enrich the project to reach targeted beneficiaries and provide staff members involved with multiple opportunities to model and witness decision-making processes of which knowledge translation is part and parcel. This does require resources and in order to scale up this approach more funding and human resources are required. This can be a limitation to this approach. Additionally, project beneficiaries were not explicitly included in the PAG or knowledge translation activities, which could be seen as a limitation. Beneficiary feedback on ongoing processes and activities was channelled through TIO and local partner staff to the PAG, however this project may have been strengthened by the presence of local organisations in these processes.

There is a need to develop different program development processes that allow for both structure and agility in intervention design. This requires an investment in human resources of project staff, as well as time and travel for face-to-face co-design practices and capacity building. This way of working is in line with the recent World Report on Vision’s [26] guidance on integrated people-centred eye care. Additionally, program development and approval processes need to be adaptable and responsive to changes as new information comes to light. Funders and development organizations can support this by allowing flexibility within design, funding approval, implementation and reporting processes. 

## 5. Conclusions

This paper documented the process of continuous knowledge translation that was undertaken when developing an intervention to reduce gender inequities in rural Nepal. The process included multiple steps including: the undertaking and interpretation of evidence from formative research; the subsequent re-design of a program intervention; a corresponding action-oriented pragmatic trial to develop, trial and adapt strategies in real time; and the inclusion of a project advisory group for on-going support, and guidance in overcoming programmatic challenges within the exploration of the trial. This articles spotlights the processes that led to the development of knowledge and the translation of evidence, and the capacity-building and strengthening of relationships with project partners, pointing to the importance of focusing on “process” and not just “outcomes” when implementing gender equity programming. The design of eye health interventions that will be effective in reaching those left behind requires a design process that builds on deep contextual knowledge, a participatory approach, and a willingness to challenge stereotypes and dive deep into the underlying issues that affect access through a gender lens.
